# Vegetable Organography; or an Analytical Description of the Organs of Plants

**Published:** 1839-07

**Authors:** 


Art. VI.—*Vegetable Organography; or an Analytical Description of the Organs of Plants.* By M. A. P. De Candolle. Translated by Boughton Kingdon. *With Plates.* Parts I.-IV.—*London,* 1839.

The celebrated work of which this is a translation is known to all botanists throughout the world as one of the most classical productions that have illustrated and advanced scientific botany. To every British botanist who has not access to the original work, the present publication will be a great boon, and we think English literature much indebted to Mr. Kingdon for the pains he has taken to make so valuable an addition to its stores. The translation seems accurate and neat; the plates are very good; and the whole publication is no less elegant than useful.

